# dCas9/CRISPR-based methylation of *O-6-methylguanine-DNA methyltransferase* enhances chemosensitivity to temozolomide in malignant glioma

**DOI:** 10.1007/s11060-023-04531-z

**Published:** 2024-01-15

**Authors:** Serendipity Zapanta Rinonos, Tie Li, Sean Thomas Pianka, Terry J. Prins, Blaine S. C. Eldred, Bryan M. Kevan, Linda M. Liau, Phioanh Leia Nghiemphu, Timothy F. Cloughesy, Albert Lai

**Affiliations:** 1 Department of Neurosurgery, Adam Michael Rosen Neuro-Oncology Laboratories, Preston A. Wells, Jr. Center for Brain Tumor Therapy, University of Florida, Gainesville, FL USA; 2 Department of Neurology, UCLA Medical Center, Los Angeles, CA USA; 3 Department of Neurosurgery, UCLA Medical Center, Los Angeles, CA USA

**Keywords:** Malignant glioma/glioblastoma (GBM), *MGMT*, Chemoresistance, Epigenetics, Methylation, CRISPR therapeutics

## Abstract

**Background:**

Malignant glioma carries a poor prognosis despite current therapeutic modalities. Standard of care therapy consists of surgical resection, fractionated radiotherapy concurrently administered with temozolomide (TMZ), a DNA-alkylating chemotherapeutic agent, followed by adjuvant TMZ. *O*-6-methylguanine-DNA methyltransferase (MGMT), a DNA repair enzyme, removes alkylated lesions from tumor DNA, thereby promoting chemoresistance. *MGMT* promoter methylation status predicts responsiveness to TMZ; patients harboring unmethylated *MGMT* (~60% of glioblastoma) have a poorer prognosis with limited treatment benefits from TMZ.

**Methods:**

Via lentiviral-mediated delivery into LN18 glioma cells, we employed deactivated Cas9-CRISPR technology to target the *MGMT* promoter and enhancer regions for methylation, as mediated by the catalytic domain of the methylation enzyme DNMT3A. Methylation patterns were examined at a clonal level in regions containing Differentially Methylation Regions (DMR1, DMR2) and the Methylation Specific PCR (MSP) region used for clinical assessment of *MGMT* methylation status. Correlative studies of genomic and transcriptomic effects of dCas9/CRISPR-based methylation were performed via *Illumina* 850K methylation array platform and bulk RNA-Seq analysis.

**Results:**

We used the dCas9/DNMT3A catalytic domain to achieve targeted *MGMT* methylation at specific CpG clusters in the vicinity of promoter, enhancer, DMRs and MSP regions. Consequently, we observed MGMT downregulation and enhanced glioma chemosensitivity in survival assays in vitro, with minimal off-target effects.

**Conclusion:**

dCas9/CRISPR is a viable method of epigenetic editing, using the DNMT3A catalytic domain. This study provides initial proof-of-principle for CRISPR technology applications in malignant glioma, laying groundwork for subsequent translational studies, with implications for future epigenetic editing-based clinical applications.

**Supplementary Information:**

The online version contains supplementary material available at 10.1007/s11060-023-04531-z.

## Introduction

For malignant glioma, specifically glioblastoma (GBM, IDH wild-type), a central aspect of clinical decision-making and prognosis is the de novo methylation status of the promoter of *O-6-methylguanine-DNA methyltransferase* (*MGMT*) [1–8], routinely assayed during neuropathologic diagnosis. *MGMT* repairs the toxic DNA lesion *O6-*methylguanine induced by alkylating chemotherapeutic agents, such as temozolomide (TMZ), thereby undermining the mechanism of action of TMZ, leading to chemoresistance. As the only FDA-approved drug with relative improvement in survival, TMZ comprises the current standard of care chemotherapy in combination with fractionated radiation [6, 7]. Approximately 40% of GBM patients harbor methylated *MGMT*, silencing expression in tumor cells, enhancing chemosensitivity and survival (progression-free survival (PFS) = 10.3 months, overall survival (OS) = 21.7 months) [2]. By contrast, the preponderance of GBM patients (60%) harbor unmethylated *MGMT* and exhibit chemoresistance to TMZ [1–3, 5–12], markedly reducing survival (PFS = 5.3 months, OS = 12.7 months) [2].

To date, multiple attempts to combat TMZ chemoresistance, via direct inhibition or cellular depletion, have yielded no significant clinical improvements. A Phase II clinical trial using the direct MGMT inhibitor *O*-6-benzylguanine showed limited benefits but lacked clinical feasibility due to severe dose-limiting toxicities (including off-target bone marrow suppression) [13, 14]. A Phase III trial implemented dose-dense TMZ, aiming to deplete MGMT in tumor cells, but failed to improve TMZ sensitivity [15]. Given these adverse findings, we employed an alternative, CRISPR-based mechanism to target *MGMT*, consisting of a chimeric fusion of deactivated Cas9 (dCas9, lacking endonuclease activity) with an epigenetic editor, DNA methyltransferase 3A catalytic domain (DNMT3A-CD); this fusion protein is hereby collectively abbreviated as “d3A”. This system enabled targeted methylation of a subset of existing CpG sites within the *MGMT* gene with the goal of decreasing MGMT expression and increasing sensitivity to TMZ, without the need for cleavage of the target gene sequence [16, 17]. This approach has multiple advantages in comparison to other gene silencing techniques: (1) *Specificity*, due to single guide RNA (sgRNA) interactions with dCas9, facilitating target gene recognition; and (2) *reversibility and relative safety*, due to preservation of the underlying target DNA sequence, resulting from deactivation of Cas9 endonuclease activity (avoiding permanent cleavage of gene sequences) and due to modification at the epigenetic level only (methylation). Furthermore, we used multiple sgRNAs to methylate a wider range of target sequences, encompassing the promoter and enhancer region, thereby enhancing effects on *MGMT* transcription. Our target regions also included: (1) Differentially Methylated Region 2 (DMR2), most highly associated with MGMT mRNA suppression [18]; and (2) Methylation-Specific Polymerase Chain Reaction (MSP) region, conventionally used clinically to determine *MGMT* methylation status, located within DMR2 [2, 19].

We hypothesized that CRISPR-mediated methylation of the *MGMT* promoter region may serve as a therapeutic strategy to enhance chemosensitivity in malignant glioma by silencing *MGMT* expression. Here we demonstrated that d3A/CRISPR-directed methylation, encompassing portions of the promoter, enhancer, DMR and MSP regions, successfully downregulated MGMT expression, significantly improved TMZ chemosensitivity via in vitro survival assays, and yielded minimal off-target effects, as per genome-wide and transcriptome-wide correlative analyses (*Illumina* EPIC 850K methylation array and bulk RNA-Seq).

## Methods

### Cell culture and treatments

LN18 cells (*ATCC*, Cat#CRL-2610) were grown in standard conditions (DMEM cell culture medium, 10% fetal bovine serum and penicillin/streptomycin). TMZ (*Santa Cruz Biotechnology* Cat #85622-93-1) was dissolved in DMSO.

### Plasmids and lentiviral transduction

dCas9-DNMT3A catalytic domain plasmids were constructed by Pflueger et al. (*Addgene*, Cat#100936). Cas9 plasmids were obtained from Addgene (Cat#108100). We designed sgRNA sequences using the Broad Institute *Genetic Perturbation Platform*. Refer to Table S1 (Supplementary Information, Online Resource 1) for input sequence and resultant sgRNA sequences.

All sgRNA constructs were mounted on lentivirus-compatible plasmids (*Vector Builder*). Plasmids were packaged with pMD2.G VSV-G envelope plasmid (*Addgene*, Cat#12259), pCMVR8.74 packaging plasmid (*Addgene*, Cat#22036), and X-tremeGENE HP DNA Transfection Reagent (*MilliporeSigma*, Cat#XTGHP-RO) in HEK293T cells cultured in DMEM; virus was harvested after 48 h. LN18 cells were transduced with lentivirus-containing media and culture media in a 1:3 ratio including polybrene (1.0 μg/mL) for 48 h, with 24-h recovery in DMEM, prior to antibiotic selection.

### Bisulfite sequencing

DNA was isolated using DNEasy Blood & Tissue Kit (*Qiagen*, Catalog #69506). Bisulfite conversion was accomplished using *EZ DNA Methylation-Gold* (*Zymo Research*, Catalog #D5005) per kit protocol. Nested PCR primers were used as follows:

- Region 1: First PCR primer pair F4/R4; second PCR pair F5/R4.
- Region 2: First PCR primer pair F1/R1; second PCR pair F1/SeqR.

Refer to Table S1. Sequencing reactions were performed using BigDye Terminator v3.1 Cycle Sequencing Kit (*ThermoFisher*, Cat#433750) with sequencing primers (R4 primer = Region 1; SeqR primer = Region 2); samples were purified by PCR Clean-Up Performa Spin Columns (*EdgeBio* Cat#13266) and submitted for Sanger sequencing analysis (*Laragen*, Culver City, CA).

### TA cloning

Sodium bisulfite-treated genomic DNA underwent nested PCR as above, for either Region 1 or 2. Resultant amplicons (second PCR products) were ligated with plasmid vector using the TA cloning kit (*New England BioLabs*, Cat#E1203S), used to transform DH5α competent *E. coli* cells (*Invitrogen*, Cat#18258012) by standard methods; clonal plasmids were isolated by PureLink™ HiPure Plasmid Miniprep kit (*Invitrogen*, Cat#K210003). DNA plasmid clones were sequenced per standard sequencing protocols as above.

### RT-qPCR

RT was performed using SuperScript™ II Reverse Transcriptase (*Invitrogen*, Cat#REF100004925), followed by qPCR using standard protocols, with *Roche* FastStart Universal SybrGreen Master (Rox) (*Sigma Aldrich*, Cat#4913850001); annealing temperature: 55 °C; cycle: 30 (see Table S1 for primer sequences).

### Methylation-specific PCR

Refer to previous publication for methods [19].

### Immunoblotting

Western blot was performed by standard protocols using primary antibodies: anti-HA, rabbit, (1:1000, *Sigma*, Cat#H6908-100mL); anti-MGMT, mouse (1:1000, *ThermoFisher*, Cat#35-7000); GAPDH, mouse (1:2000, *Proteintech* Cat#60004-I-Ig) (see Online Resource 1 (Supplementary Information) for further details).

### Cell survival assays

MTT [3-(4,5-dimethyl-2-thiazolyl)-2,5-diphenyl-2*H*-tetrazolium bromide] assays were performed using standard protocols. Briefly, a uniform number of cells (2300 or 4600 per plate) were cultured in 24-well plates for 5 days with TMZ (100 μM or 250 μM) or DMSO control treatment, exposed to pre-mixed MTT solution (0.5 mg/mL in culture media), and incubated at 37 °C for 1 h. Formazan product was extracted by cell lysis with DMSO (300 μL) and measured (560 nm absorbance with background subtraction of 660 nm). Clonogenic assays were conducted as previously described. Cells (250 or 350 per well) were seeded in 60-mm plates. After overnight incubation, TMZ/DMSO was added and replaced after 6 days. Following 12 days of total treatment, cells were washed in phosphate-buffered saline (PBS), fixed in 100% methanol, and stained with 0.5% crystal violet/25% methanol solution.

### Statistical methods for MTT and clonogenic assays

Data were analyzed in Prism 9 via a student’s *t*-test or analysis of variance (ANOVA) where appropriate, and ANOVA post hoc analyses were performed using Tukey’s HSD test for multiple comparisons.

### Differential methylation analysis and transcriptomic analysis

Genome-wide methylation and transcription profiling was achieved via the *Illumina* platform EPIC 850K methylation array. Briefly, genomic data (IDAT files) were imported into *R* [23] and processed via the *minfi* package for the generation of methylation M-values [24]. Custom scripts were written to determine variance (standard deviation) for each probe for unsupervised hierarchical clustering, while supervised hierarchical clustering was achieved by fitting the data to a linear model and evaluating it via empirical Bayes for differential methylation [25]. Expression (RNA transcription) was determined by aligning bulk RNA-Seq data to the genome via *minimap2* and counting genes via *HTSeq*, which were then analyzed for differential expression using *DESeq2* [26]. Theoretical sgRNA “hits” were determined using the Off-Spotter platform [27] (see Online Resource 1 (Supplementary Information) for further details).

## Results

### Selection of unmethylated *MGMT* cellular background, epigenetic editor, sgRNA construct design, and expression verification

**Fig. 1:**
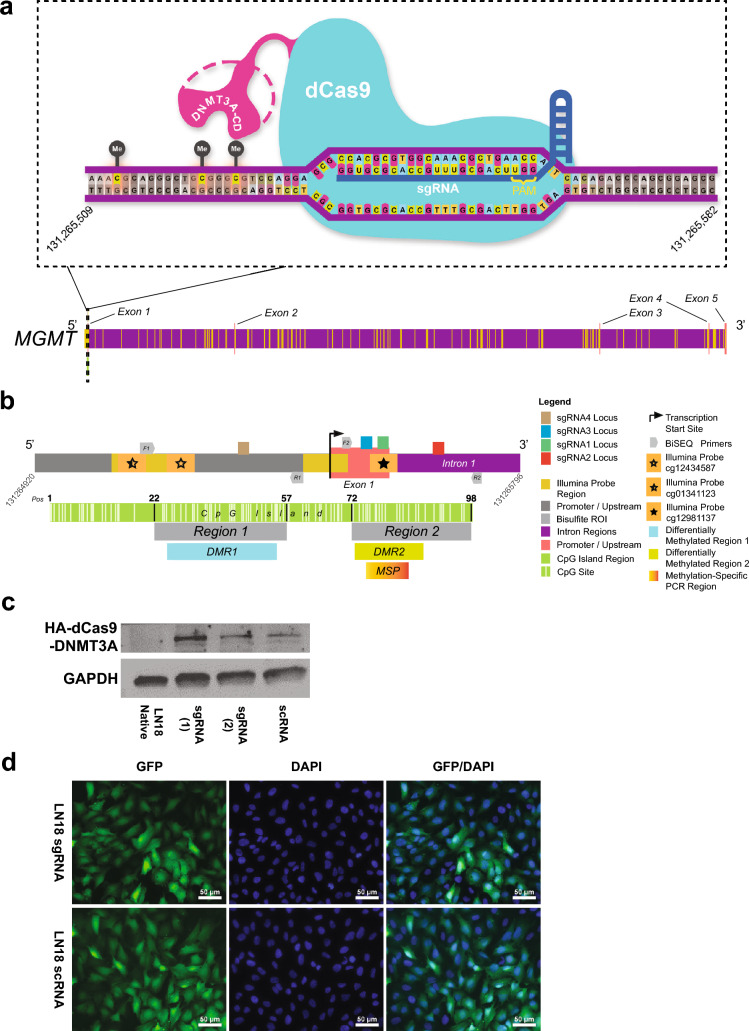
Schematic overview and verification of the dCas9/DNMT3A catalytic domain CRISPR-based methylation system in LN18 human glioma cells. **a** Schematic representation of the dCas9-DNMT3A-CD complex bound to a segment of DNA at the *MGMT* gene on chromosome 10. Deactivated Cas9 (dCas9) is unable to cut DNA and is fused to the DNMT3A catalytic domain. Single guide RNA (sgRNA) sequences (blue) can bind to complementary sequences within the genome, which permits dCas9 (teal) to be able to recognize and bind to DNA (purple). Once bound, DNMT3A-CD (magenta) can then induce methylation in CpG sites (represented as the “Me” labeled black circles) upstream of sgRNA complementary sequences. **b** Map of *MGMT* CpG island, 762 bp in length, encompassing promoter, exon 1, enhancer, and intron 1 regions. Comprehensive map of the *MGMT* gene with superimposed locations of *Illumina* probes (yellow boxes) as well as exon (salmon), intron (purple), and promoter/upstream (gray) regions. Locations of complementary sequences to the four sgRNAs are as shown. Open star, half-closed star and closed star regions indicate locations of differentially methylated *Illumina* probes. F1/R1 and F2/R2 indicate nested PCR primer pairs for Region 1 and Region 2, respectively. Inset: *MGMT* CpG island (individual CpG sites in light green) and relative locations of Region 1 and Region 2. CpG sites 22, 57, 72, and 96 indicate the specific sites flanking each region, numbered in order from 5′ to 3′ within the CpG island. Differentially methylated regions are shown (DMR1 and DMR2), located within assayed Region 1 and 2, respectively. MSP region is also shown within DMR2. Genetic regions and positions on Chromosome 10 were determined using the UCSC genome browser (GRCh37/hg19 assembly) and the 850K array probe annotation file provided by *Illumina*. **c** Verification of dCas9-DNMT3A-CD (d3A) protein expression in LN18 human glioma cells. Western blot images of HA-tagged d3A fusion protein in LN18 cells sequentially transduced with pLVP-dCas9-DNMT3A-CD-V2 and pLenti-sgRNA-GFP (versus native cell line as negative control with no transduced constructs); representative blot shown here (from at least three replicate experiments). “scRNA” indicates scrambled sgRNA transduction; “sgRNA(1)” and “sgRNA(2)” indicate replicate samples derived from cells with MGMT-sgRNA 1, 2, 3 and 4 transduction. Expected size of the d3A fusion protein is approximately in the 200 kDa range, as shown, using anti-HA antibody-mediated detection. GAPDH served as the loading control. **d** Verification of sgRNA-GFP lentiviral transduction in LN18 human glioma cells. Representative fluorescent microscopic images (×40 magnification) of the same LN18 cell lines in part **c** demonstrating GFP signal detection in cells transduced with GFP-tagged pLenti-sgRNA (LN18sgRNA1, 2, 3, 4) vs. the scrambled sgRNA GFP-tagged pLenti-scRNA (LN18 scRNA). DAPI shown as nuclear stain, with merged images in far-right column. Scale bar as shown (50 μm)

After screening multiple glioma cell lines with MSP, western blot analysis and bisulfite sequencing (BiSEQ), we selected LN18 human glioma cells, given the unmethylated *MGMT* status within target regions of interest and high levels of de novo MGMT expression (Fig. S1). LN18 exhibits high TMZ EC_50_ values (400 μM) amongst glioma cell lines with chemoresistance reported over time [28]. For the effector enzyme, we selected the de novo methylator DNMT3A catalytic domain fused to dCas9 via a flexible linker plus HA tag [17], comprising a smaller plasmid construct more amenable to lentiviral delivery methods (Fig. 1a).

Given reported enhanced efficiency of d3A-mediated methylation using multiple sgRNAs broadening target regions [16, 17, 29–32], we designed four sgRNAs with specific homology to *MGMT* regions encompassing promoter, enhancer and exon 1 regions, within a GFP-tagged lentiviral plasmid (Fig. 1a, b). As a negative control, we established LN18 cells expressing d3A plus scRNA (scrambled sgRNA), bearing no exact sequence homology to any mammalian genomic regions (see Online Resource 1). We verified expression of HA-tagged d3A via western blot analysis of sgRNA-transduced cells and scRNA-transduced cells, relative to baseline native LN18 cells (Fig. 1c). Fluorescent microscopy confirmed sgRNA and scRNA expression (Fig. 1d), showing efficient lentiviral transduction efficiency (over 90% cells exhibiting GFP positivity).

### BiSEQ confirms targeted *MGMT* methylation via dCas9/CRISPR system, suggesting methylation hotspot locations and possible minimum radius of methylation

**Fig. 2:**
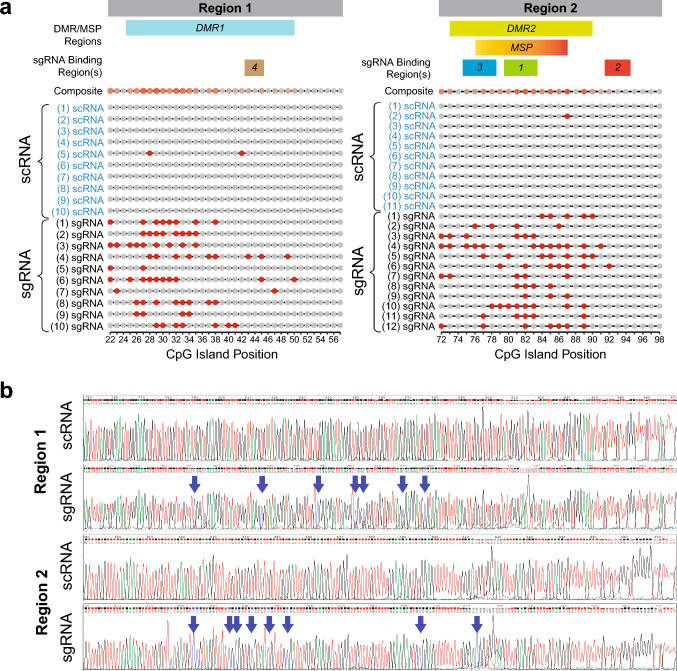
Bisulfite sequencing of representative clones with induced methylation patterns of CpG sites at CRISPR-targeted regions within the *MGMT* CpG island. **a** Lollipop schematic illustrating the distribution of CpG methylation sites in each representative clonal population (red circle = methylated site; gray circle = unmethylated site). Genomic DNA from LN18 cells containing either d3A/scRNA or d3A/sgRNA underwent sodium bisulfite treatment, followed by nested PCR amplification. Region 1 contains the target binding region for sgRNA 4 (as indicated by “4”); Region 2 contains the target binding regions for sgRNA 1, 2, and 3 (as indicated by “1”, “2” and “3”, respectively). Region 1 contains DMR1; Region 2 contains DMR2 and the MSP region. Amplicons of Region 1 and Region 2 were obtained separately and used to generate individual clones for each region via TA cloning. Abbreviations for individual clone nomenclature: “scRNA” indicates clones transduced with scrambled sgRNA; “sgRNA” indicates clones transduced with sgRNA1, 2, 3, and 4. Numbers preceding “scRNA” or “sgRNA” designation indicate individual clone numbers. Each row of the schematic (per category, scRNA or sgRNA) represents a single clone. The composite row displays a summary of the relative frequencies of methylation at each CpG site amongst the assayed clones. Regional CpG sites as per Malley et al. [18]: DMR1 = CpG 25–50; DMR2 = CpG 73–90; MSP = CpG 76–87 (MSP-F = CpG 76–80; MSP-R = CpG 84–87). Core/minimal promoter = CpG 50–62; Enhancer = CpG 82–87. Refer to Table S2 (Supplementary Information, Online Resource 2) for details. **b** BiSEQ chromatograms of representative clones, demonstrating methylation of target DNA sequences within the MGMT CpG island, derived from LN18 cells transduced with dCas9/DNMT3A-CD plus sgRNA constructs. Blue arrows indicate CpG sites that are methylated (converted from T-G to C-G). Region 1 includes the sequence targeted by sgRNA4 (4). Region 2 includes the sequence targeted by sgRNA 1, 2 and 3 (1, 2, 3)

Using BiSEQ, the gold standard for methylation status confirmation of individual CpG sites, we analyzed the following targets separately using TA cloning methods: (1) “Region 1,” encompassing sgRNA4, core/minimal promoter, and promoter regions; (2) “Region 2,” comprised of exon 1, intron 1 and enhancer in proximity to sgRNA1, 2, and 3 (Figs. 1b, 2a). Due to aforementioned key components which influence gene transcription, we selected these target regions, which also included Differentially Methylated Regions (DMR1 and DMR2) and the MSP region [18, 19]. Representative chromatograms are shown (Fig. 2b), comparing scRNA vs. sgRNA clones; blue arrows indicate methylated CpG sites (retained cytosines at CpG sites, “CG”) vs. unmethylated CpG sites (converted to thymine, “TG”).

The “composite” row of the schematic (Fig. 2a) illustrates relative frequency of methylated CpG sites; red shading intensity indicates methylation frequency amongst the clones analyzed here. Amongst Region 1 clones, there is apparent asymmetry with respect to methylation occurrence: The preponderance of methylated sites were located in the 5′ upstream region relative to sgRNA4, at CpG 22–38 (DMR1 and promoter region) but minimally noted in the partially overlapping region with the core promoter (CpG 50 and downstream to end of Region 1), which is downstream from sgRNA4. For Region 2, which encompassed sgRNA3, sgRNA1, and sgRNA2 target sequences, methylated CpG sites clustered toward the center of the amplicon, upstream of sgRNA2, within DMR2 and MSP regions, with greatest density and frequency at CpG 77, 81–89. Within Region 1, methylation appears to extend to a 20-bp radius upstream from sgRNA4, to the upstream 5′ limit of the amplicon (CpG 22). It is unknown whether methylation extends further upstream beyond CpG 22, as we did not assay this region here. Given close proximities of multiple sgRNA target sequences within Region 2 (sgRNA 1, 2, 3), methylation radius cannot be reliably ascertained in Region 2.

### CRISPR-based *MGMT* methylation is sufficient to reduce MGMT expression and enhance chemosensitivity

**Fig. 3:**
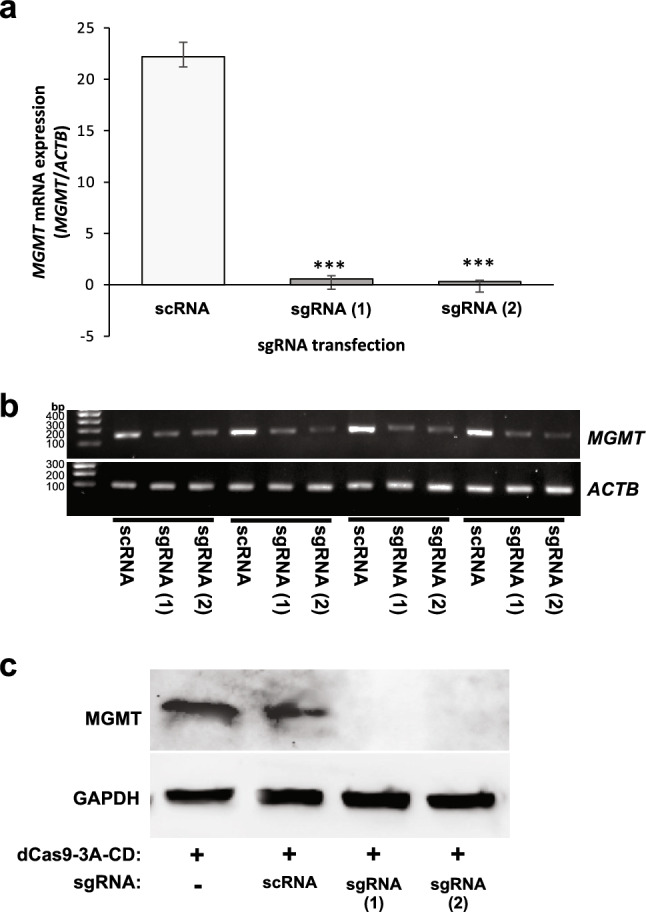
Expression of MGMT mRNA and protein in LN18 human glioma cells with CRISPR-mediated MGMT methylation via dCas9-DNMT3A-CD and *MGMT-*specific sgRNAs. **a** MGMT mRNA expression detected by RT-qPCR in LN18 cells expressing d3A/sgRNA targeted towards *MGMT*. Top: qPCR bar figure of expression mean ± SEM (from a total of four repeated experiments). **b** DNA gel electrophoresis images of corresponding qPCR for *MGMT* end products (from four separate experiments). *Actin B* (*ACTB*) served as the internal control. **c** Western blot analysis of MGMT expression levels in LN18 cells. GAPDH was used as the loading control. Representative results are shown here (from a total of four replicate experiments for scRNA and sgRNA-expressing lysates); additional negative control included in this blot is protein lysate from LN18 cells expressing dCas9-DNMT3A-CD fusion protein only, without scRNA or sgRNA construct (first lane). Abbreviations: *scRNA* = cells transduced with scrambled sgRNA, *sgRNA* = cells transduced with sgRNA 1, 2, 3 and 4. (1), (2) = replicate samples

Using polyclonal populations of cell lines stably transduced with *MGMT*-specific sgRNAs (sgRNA) vs. scrambled sgRNA (scRNA), RT-qPCR was performed, revealing significant downregulation in MGMT mRNA expression (p < 0.001, Fig. 3a). Gel electrophoresis of *MGMT* PCR end products is also shown, with *Actin B (ACTB) as the internal control* (Fig. 3b). Protein lysates from the same cell lines were analyzed by western blot, revealing marked downregulation of MGMT protein expression in the context of CRISPR-based methylation (Fig. 3c).

**Fig. 4:**
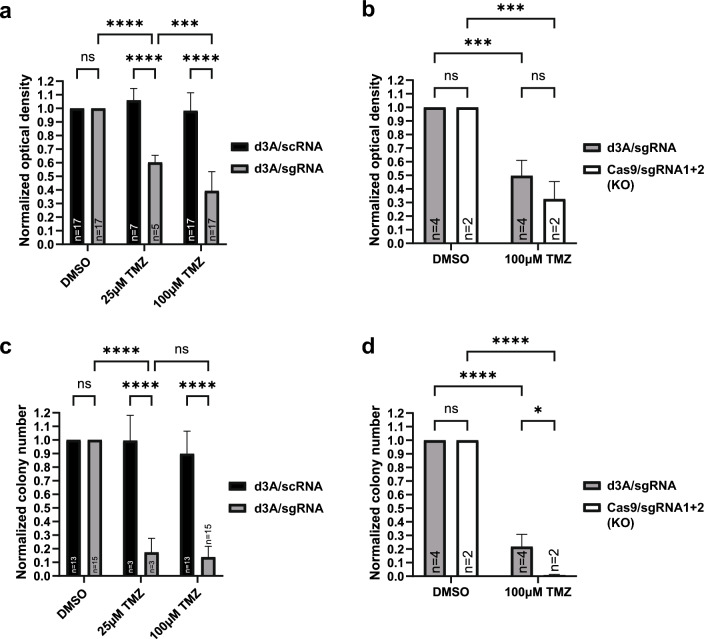
Effects of dCas9-DNMT3A-CD/*MGMT*-specific sgRNA targeted methylation on the sensitivity of glioma cells to TMZ. MTT and clonogenic assays demonstrate effects of d3A/sgRNA CRISPR-based methylation on the survival of LN18 glioma cells treated with TMZ in vitro. The “n” indicates the number of separate experiments performed for each cell type. Results were normalized to the average negative control treatment condition (drug vehicle DMSO). TMZ concentrations ranged from 25 to 100 μM. Horizontal bars for all figures represent post hoc Tukey’s multiple comparisons test results for groups of interest following two-way ANOVA (“ns” indicates “p > 0.05”; * indicates p ≤ 0.05; ** indicates p ≤ 0.01; *** indicates p ≤ 0.001; **** indicates p ≤ 0.0001). Black bars = d3A/scRNA cells (negative control); gray bars = d3A/sgRNA cells (with full sgRNA constructs 1, 2, 3, and 4); white bars = Cas9/sgRNA1 + 2 cells (KO) (*MGMT* knockout via Cas9, with sgRNA constructs 1 and 2). **a **TMZ sensitivity measured via MTT survival assays in LN18 cells co-expressing d3A and *MGMT*-targeting sgRNAs compared to scRNA. Differences were observed between sgRNA treated cells (ANOVA, F(1, 74) = 213.3, p < 0.0001) and applied TMZ concentration (ANOVA, F(2, 74) = 93.52, p < 0.0001); an interaction was also observed between sgRNA/scRNA and TMZ (ANOVA, F(2, 74) = 86.92, p < 0.0001). MTT cell survival was calculated by subtracting 560 nm readings from 550 nm readings for TMZ-treated cells (25 and 100 μM, 5 days) versus DMSO treatment control condition, followed by normalization to the average control condition. **b **Effect of sgRNA methylation-mediated TMZ sensitivity (ANOVA, F(1, 8) = 134.9, p < 0.0001) was comparable between dCas9-DNMT3A-CD vs. Cas9-mediated *MGMT *knockout (KO) cells (ANOVA, F(1, 8) = 2.843, p = 0.1303) when evaluated via MTT survival assays. **c** Clonogenic assay revealed TMZ sensitivity results comparable to MTT (ANOVA, F(2, 56) = 186.2, p < 0.0001). Two-way ANOVA results revealed an effect of sgRNA treatment (F(1, 56) = 287.5, p < 0.0001) as well as an interaction between TMZ sensitivity and sgRNA treatment (F(2, 56) = 124.8, p < 0.0001). **d **Clonogenic assay demonstrated similar results to **b** with increased TMZ sensitivity in dCas9-DNMT3A-CD and Cas9-mediated *MGMT *knockout (KO) cells (ANOVA, F(1, 8) = 685.1, p < 0.0001). Two-way ANOVA also showed a difference between the dCas9 and Cas9 systems (F(1, 8) = 9.587, p = 0.0147) as well as an interaction between system and TMZ sensitivity (F(1, 8) = 9.587, p = 0.0147)

We investigated whether CRISPR-mediated epigenetic conversion was sufficient to enhance TMZ chemosensitivity, using MTT and clonogenic survival assays. We compared survival in sgRNA vs. scRNA cells treated with DMSO (vehicle), 25 μM TMZ, or 100 μM TMZ using two-factor ANOVA. ANOVA analysis of MTT results revealed main effects of sgRNA treatment (F(1, 74) = 213.3, p < 0.0001), TMZ treatment (F(2,74) = 93.52, p < 0.0001), as well as an sgRNA/TMZ interaction (F(2, 74) = 86.92, p < 0.0001) (Fig. 4a). Clonogenic assays yielded analogous results between low-dose TMZ-treated cells and DMSO controls (ANOVA, TMZ treatment F(2, 56) = 186.2, p < 0.0001) (Fig. 4c). A post hoc Tukey test (p < 0.0001) showed enhanced sensitization to TMZ occurred at concentrations as low as 25 μM (Fig. 4c).

To compare direct Cas9 endonuclease disruption of *MGMT* vs. deactivated Cas9-based methylation effects, we generated Cas9-mediated *MGMT* knockout LN18 cells (transduced with sgRNA constructs 1 and 2). Extent of chemosensitization was comparable between Cas9-mediated knockout lines vs. d3A-mediated methylation when evaluated via MTT (ANOVA, F(1, 8) = 2.843, p = 0.1303), but a difference was observed via clonogenic assays (F(1, 8) = 9.587, p = 0.0147). Both assays, however, demonstrate clear TMZ sensitivity due to d3A-mediated methylation (Fig. 4b, d). Essentially no TMZ sensitivity was observed in LN18 d3A cells expressing the scRNA negative control construct. This indicates that epigenetic conversion is a sufficient, viable alternative strategy to enhance chemotherapeutic response, obviating the need for permanent target gene cleavage.

### Validation of dCas9/CRISPR-based target specificity: Genome-wide vs. transcriptome-wide correlative analysis

**Fig. 5:**
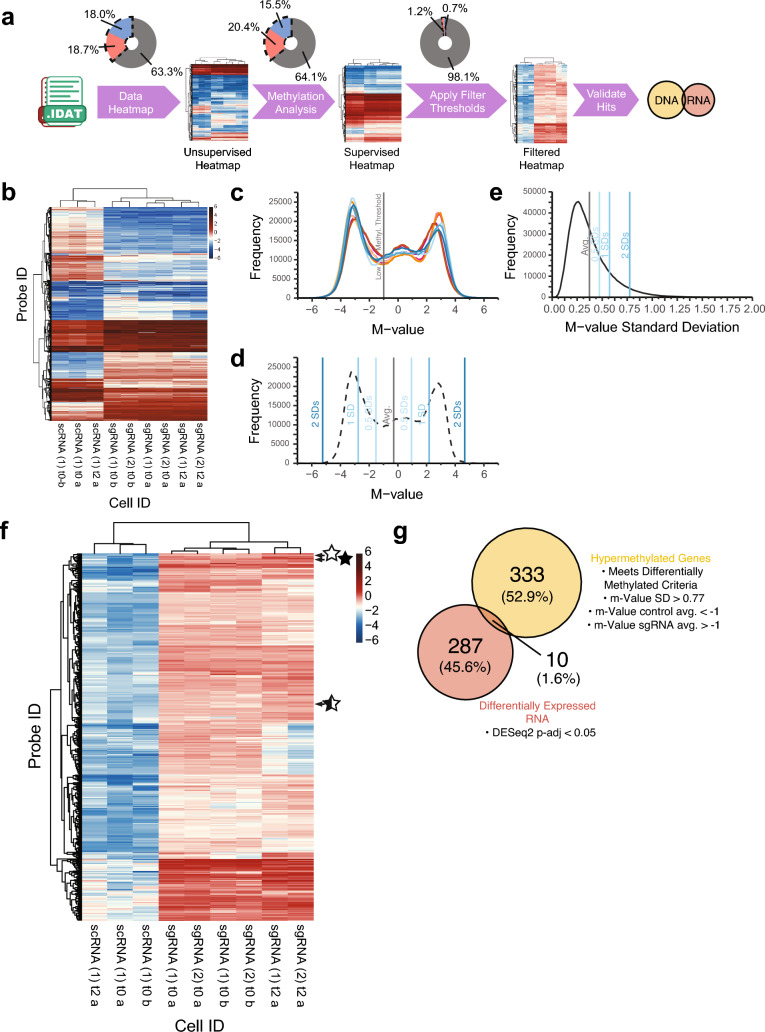
Validation of CRISPR-based dCas9-DNMT3A-CD targeted *MGMT* hypermethylation and differential RNA expression via correlation of *Illumina* EPIC 850K methylation array and RNA-Seq analysis. **a** Overview of the *Illumina* pipeline and generation of supervised hierarchical heatmaps. Raw data from the *Illumina* array (.idat files) were imported into R and matched to the *Illumina* annotation manifest by probe. Methylation values by probe were passed through a quality control check, CpG sites with single nucleotide polymorphisms (SNPs) were removed, and the data was normalized. These data were then clustered for methylation state by sample and probe ID using M-values with high variance (>2.5 SDs), for unsupervised heatmap generation. Probes were further isolated by adjusted p-value < 0.05 (linear fit and eBayes analysis) for supervised heatmap generation. “On Target” and “Off Target” probes were identified by further filtering the supervised differential methylation data by M-values with high variance across samples (>2 SDs) and a low control average M-value (<−1) and high sgRNA average M-value (>−1). These genes were cross referenced with bulk RNA-Seq differential expression data (evaluated via DESeq2) to determine functionally significant “On Target” and “Off Target” hits. Each step shows a donut plot of approximate percentage of genes from the total array that emerged from the filter criteria for that step, with hypermethylation shown in red, hypomethylation shown in blue, and no change (under that criterion) shown in gray. **b** Unsupervised hierarchical clustering of M-values by *Illumina* probe (rows) and LN18 cell treatment (columns). M-value variance (standard deviation) across cell type for each probe was calculated for the entire *Illumina* 850K array and probes with the highest level of variance (2.5 SDs > average M-value SD; N = 21,278) were isolated and plotted as a heatmap. *Definition of nomenclature*: (1), (2) = replicates; t0 = baseline harvest time point (corresponds to approximately 2 weeks after final lentiviral transduction, in this case, s/p GFP-sgRNA or GFP-scRNA transduction); t2 = harvested 2 months after t0; a = indicates samples run on first array batch; b = indicates samples run on second array batch, subsequent to first array. (We performed two separate arrays, at different times, distinguished here by a and b.) **c** Raw M-value distributions for all *Illumina* probes and cell samples. Control LN18 scRNA samples are shown by the blue traces (scRNA (1) t0 a, traces scRNA (1) t2 a, traces scRNA (1) t0 b) traces, while the LN18 sgRNA samples are shown by the magenta traces (sgRNA) (1) t0 a, (sgRNA) (1) t2 a, (sgRNA) (1) t0 b) and orange traces (sgRNA) (2) t0 a, (sgRNA) (2) t2 a, (sgRNA) (2) t0 b) traces. The approximate cutoff point for the first peak and “low methylation” threshold is indicated by the vertical gray line (−1). **d** Average trace of all control LN18 NSC samples, with the average M-value across all probes indicated by the vertical gray line. Vertical blue lines represent M-value standard deviations of varying degrees above and below this average. **e** Distribution of M-value variance for all *Illumina* probes across all samples, with summary statistics similar to panel **d** superimposed. These M-value distribution plots were used for establishing thresholds for determining large increases in methylation state between control LN18 scRNA and sgRNA samples. **f** Following validation via unsupervised hierarchical clustering and generation of an initial supervised heatmap (refer to pipeline in **a** and Methods), we applied additional filtering for hierarchical clustering of all CpG island probes found to be differentially methylated according to the following criteria: (1) p-adj < 0.05, (2) found in CpG island region, (3) exhibited an increase in methylation M-value from control LN18 scRNA to sgRNA cells greater than 2 standard deviations above the average M-value variance (SD) in all cells/probes, and (4) contained an average control LN18 scRNA methylation M-value of less than −1. Of these criteria, three probes within the *MGMT* gene were identified: cg12434587 (open star), cg01341123 (half-closed star), and cg12981137 (closed star), all of which were near sgRNA loci (see Fig. 1). **g** Of all the probes surveyed in **a**, 333 unique genes were identified and intersected with *DESeq2* differential expression bulk RNA-Seq data (Wald test, p-adj < 0.05). Genes found in both data sets were deemed to be “On-Target and Off-Target” effects, which included *MGMT* and nine other genes (refer to Results main text for details)

To determine on-target and off-target effects of dCas9/CRISPR-based methylation, we performed genome-wide analysis of LN18 cells expressing the d3A/sgRNA system vs. d3A/scRNA negative control using the *Illumina* EPIC 850K methylation array, followed by transcriptomic analysis via bulk RNA-Seq (pipeline shown in Fig. 5a). Probes with the highest M-value variances (2.5 SDs greater than the average M-value SD) were used to generate an unsupervised hierarchical heatmap (total 21,278 probes), plotted by *Illumina* probe and LN18 cell type (sgRNA vs. scRNA). This necessary step demonstrated that samples of concordant cell type segregated accordingly (scRNA samples clustered together and sgRNA samples likewise clustered together) (Fig. 5b). Supervised hierarchical clustering was performed sequentially (details in Methods and Supplementary Information/Online Resource 1), identifying genes with the greatest difference in methylation state following d3A/CRISPR-mediated methylation (baseline unmethylated in scRNA cells but methylated in sgRNA cells). A total of 333 unique genes were identified, including three probes within the *MGMT* gene (Fig. 5f): cg12434587 (open star) and cg01341123 (half-closed star), both in proximity to sgRNA4, and cg12981137 (closed star), in proximity to sgRNA1, 2, and 3 loci (Fig. 1b), providing additional confirmation of on-target effects.

To elucidate effects on transcriptomic changes, we performed Bulk RNA-Seq analysis on the same LN18 glioma cell lines (refer to Methods and Online Resource 1). The 333 unique genes identified by secondary supervised hierarchical clustering were cross-referenced with bulk RNA-Seq differential expression data, yielding ten total gene hits, including *MGMT* as the top probe hit, serving as on-target confirmation (Fig. 5g). Subsequently, we used the Off-Spotter program [27] to blast sgRNA 1, 2, 3, 4 sequences for off-target prediction hits. Of the ten genes that emerged from the three-part analysis, namely, (1) DNA differential methylation, (2) RNA differential expression, (3) Off-spotter intersection, *MGMT* emerged as the singular gene hit fitting all criteria; the only hits emerging were *MGMT *probes (refer to Online Resource 3, Table S3). The nine additional “off target” gene probe hits had Off-Spotter hits hundreds of thousands of bases away from the probe that emerged from analysis. One exception was *FAM84A*, but RNA expression was incongruent with DNA methylation (i.e., increased DNA methylation but with increased RNA transcription). From basic interrogation of Off-Spotter hits within 1000 bases of the *Illumina* probe, none of the results fit the aforementioned DNA/RNA criteria. Our findings suggest CRISPR-based methylation using d3A appears specific for *MGMT *with minimal off-target effects.

We also performed gene ontology analysis, using Metascape, on the differentially expressed genes following CRISPR-mediated *MGMT* methylation, from data obtained via bulk RNA-Seq. Two separate analyses were performed: One for downregulated genes (defined as DESeq2 log2 fold change less than zero) and a separate analysis for upregulated genes (defined as DESeq2 log2 fold change greater than zero). Results are shown in Table S3. Among the top categories of downregulated genes were cytokine/inflammatory signaling pathways such as NF-kB survival signaling, interleukin-10 signaling, lipopolysaccharide response, as well as cell migration positive regulatory pathways. Among the top categories of upregulated genes included adhesion, migration, chemotaxis, and extracellular matrix organization pathways. Whether these pathways exhibit any functional biologic consequences as a result of *MGMT* methylation remains to be determined.

## Discussion

The current study provides proof-of-principle evidence that the d3A/CRISPR methylation system is sufficient for targeted methylation at a high frequency and density within the *MGMT* promoter and enhancer regions (including DMR1, DMR2 and MSP). This methylation is likewise sufficient for MGMT downregulation and chemosensitization, reflected by significant reductions of tumor cell survival in vitro. Correlative analyses of genomic and transcriptomic changes provided initial validation of target specificity, with no definitive off-target effects identified. Supervised hierarchical clustering of *Illumina* methylation data identified three differentially methylated probes within *MGMT*: Two probes (cg12434587 and cg12981137) were congruent with prior reports in GBM patient samples using the MGMT-STP27 logistic regression model [33], localized to the promoter, correlating with patient outcomes; the third probe (cg01341123), upstream to Region 1, has not been previously reported as a survival correlate. Future studies can confirm epigenetic and clinical significance of this upstream region via clonal analysis. The regression model noted CpG sites in proximity to TSS and on the far 5′ and 3′ ends of the CpG island did not correlate with OS [33]. We examined the TSS region and far 3′ end, outside the regions of high methylation frequency/density, suggesting methylation in these areas is not required for MGMT suppression and chemosensitization.

Given proximity of multiple sgRNAs in Region 2, it is unknown whether they equally or hierarchically influence methylation patterns. Future studies can elucidate effects of a singular sgRNA on CpG cluster methylation, i.e. whether an upstream methylation propensity truly exists, relative to sgRNA target locus. The poorly defined methylation radius in Region 2 can be clarified with this method. The d3A/CRISPR system can be conveniently harnessed to probe relative clinical significance of CpG site methylation throughout the CpG island, effects on gene transcription, and elucidate possible methylation interdependence between CpG sites. For future clinical application, the minimum complement of sgRNA payload required to achieve the current methylation patterns should be determined.

Furthermore, as regards interpretation of the current hierarchical clustering methylation heatmaps, three main concepts are important to consider: (1) methylated probes do not necessarily correspond to transcriptional changes; (2) indirect effects on methylation cannot be disregarded; (3) multiple probes can often represent a single gene. There are numerous *Illumina* probes found across a given CpG island of a single gene, by virtue of CpG island length. Each probe, when methylated, may not translate directly into a transcriptional change, as the impact of methylation depends upon probe location, relative to the promoter/enhancer region(s) of a given gene. Future studies are needed to address these relative proximities. In addition, we cannot rule out indirect effects on methylation and transcription. MGMT is a critical DNA repair enzyme; therefore, silencing its activity may disrupt methylation indirectly. For example, in the instance that a DNA demethylase gene is not repaired in the absence of *MGMT*, resulting in failure of demethylation in a downstream target gene, this would be considered an “off-target” effect unrelated to the sgRNA/dCas9-DNMT3A system. It is currently unknown what potential gene networks might be affected by the dCas9-DNMT3A-CD/*MGMT* system through other mechanisms. However, despite this, our current methylation “off-target hits” did *not* correspond to a functional change in mRNA transcription from the DESeq2 intersection analysis, and therefore may not have a clinical impact. Further study may be warranted to ensure this is the case. Finally, although at first glance, the methylation hits may appear as a myriad, it is important to note that a single gene can be represented by multiple probes (rows) on the heatmap. For instance, *MGMT* is represented by three rows (denoted by stars in Fig. 5f); the heatmap shown here appears to be many genes, but in reality, there are few, due to this phenomenon. The vast majority of genes analyzed through the *Illumina* pipeline were unaltered. Likewise, as regards interpretation of gene ontology analysis of upregulated vs. downregulated pathways in the context of CRISPR-mediated *MGMT* methylation, it is unclear whether the aforementioned cytokine/inflammatory and chemotaxis phenomena are truly altered in this context; further studies are needed to ascertain whether these pathways exhibit any biologic effects or are simply artifactual and indirect.

During final submission preparations of the current manuscript, another manuscript was recently published [34] in which a very similar CRISPR-based approach was successfully used to sensitive LN18 human GBM cells to TMZ. As in the current study, a deactivated Cas9 system was used, in fusion with a DNMT3A-based methylation enzyme system, although with some variations in methodology. Our results support and extend these findings, reinforcing the resultant chemosensitization observed after targeted CRISPR-based methylation, in addition to specific CpG site methylation and potential off-target effect analysis at a genome-wide level.

We are optimizing conditions for CRISPR-based methylation in patient-derived gliomasphere cell lines and ex vivo xenograft studies. LN18 glioma cells provided an appropriate genetic landscape for current proof-of-principle studies, but our confirmed lack of intracranial tumor engraftment in vivo (data not shown), corroborated by previous attempts by others [35] necessitates using alternative cellular backgrounds.

Future translational studies of CRISPR-based methylation should address the following anticipated challenges: Selection/optimization of delivery vehicle (e.g., viral vectors vs. nanoparticles, and systemic vs. intratumoral delivery); payload definition (e.g., ribonucleoprotein complex of dCas9 and target sgRNAs); physiologic obstacles impacting optimal delivery (blood brain barrier impedance/penetration, solid tumor context preventing uniform penetration, and engineering mechanisms to achieve specificity for target cells). In summary, the current approach provides the initial foundation for subsequent preclinical and translational endeavors using modified dCas9/CRISPR-based epigenetic editing in the malignant glioma context, achieving chemosensitivity within a theoretically reasonable safety profile.

### Supplementary Information

Below is the link to the electronic supplementary material.

Supplementary file1 (DOCX 1573 KB)

Supplementary file2 (XLSX 18 KB)

Supplementary file3 (XLSX 18 KB)

## Data Availability

The data that support the findings of this study are available from the corresponding author upon reasonable request.
