# Supplementary material for: dCas9/CRISPR-based methylation of O-6-methylguanine-DNA methyltransferase enhances chemosensitivity to temozolomide in malignant glioma
Source: J Neurooncol. 2024 Jan 15;166(1):129–42. doi: 10.1007/s11060-023-04531-z (PMC10824881; doi:10.1007/s11060-023-04531-z)
Supplement: Supplementary file 1 — Supplementary file1 (DOCX 1573 KB) [file 11060_2023_4531_MOESM1_ESM.docx]

**SUPPLEMENTARY INFORMATION: Table S1, Fig. S1, Fig. S2, Fig. S3, Additional Methods**

**dCas9/CRISPR-Based Methylation of**

***O6-Methylguanine DNA Methyltransferase***

**Enhances Chemosensitivity to Temozolomide in Malignant Glioma**

Serendipity **Zapanta Rinonos**^2^*, Tie **Li**^1^*, Sean Thomas **Pianka**^1^*,
Terry J. **Prins**^1^, Blaine S.C. **Eldred**^1^,
Bryan M. **Kevan**^1^, Linda M. **Liau**^3^, Phioanh Leia **Nghiemphu**^1^,

Timothy F. **Cloughesy**^1^, Albert **Lai**^1^**

Author Affiliations:

^1^Department of Neurology, UCLA Medical Center, Los Angeles, CA, USA

^2^Department of Neurosurgery, Adam Michael Rosen Neuro-Oncology Laboratories,

Preston A. Wells, Jr. Center for Brain Tumor Therapy,

University of Florida, Gainesville, FL, USA

^3^Department of Neurosurgery, UCLA Medical Center, Los Angeles, CA, USA

 
**Co-first authors (Zapanta Rinonos, Li, Pianka): These authors contributed equally to this work.*
***Corresponding author:* [*AlbertLai@mednet.ucla.edu*](mailto:AlbertLai@mednet.ucla.edu)

**Table S1 Primers used in this study**

| **Description** | **Sequence** |
| --- | --- |
| Input sequence mapping to the *MGMT* region of interest (used for sgRNA design on the  Broad Institute  [Genetic Perturbation Platform](https://portals.broadinstitute.org/gpp/public/analysis-tools/sgrna-design) | TGCCCCTCGGCCCCGCCCCCGCGCCCCGGATATGCTGGGACAGCCCGCGCCCCTAGAACGCTTTGCGTCCCGACGCCCGCAGGTCCTCGCGGTGCGCACCGTTTGCGACTTGGTGAGTGTCTGGGTCGCCTCGCTCCCGGAAGAGTGCGGAGCTCTCCCTCGGGACGGTGGCAGCCTCGAGTGGTCCTGCAGGCGCCCTCACTTCGCCGTCGGGTGTGGGGCCGCCCTGACCCCCACCCATCCCGGGCGA |
| sgRNA1 | 5’–GGTGCGCACCGTTTGCGACT–3’, PAM = TGG |
| sgRNA2 | 5’–AGGCGCCCTCACTTCGCCGT–3’, PAM = CGG |
| sgRNA3 | 5’ –CTTTGCGTCCCGACGCCCGC–3’, PAM = AGG |
| sgRNA4 | 5’–AGGGCATGCGCCGACCCGGT–3’, PAM = CGG |
| Scrambled sgRNA(scRNA) | 5’ – GTATTACTGATATTGGTGGG – 3’ |
| Nested PCR primers | F1: 5’-GGATATGCTGGGACAGCC-3’  R1: 5’-GGGCCTGGGGTTCCTGG-3’  SeqR: 5’-CACCTAAAAAACACTTAAAAC-3’  F4: 5’-TTATTTGGTAAATTAAGGTATAGA-3’  F5: 5’-TGGTAAATTAAGGTATAGAGTTTTAGG-3’  R4: 5’**-**AAAACCTAAAAAAAACAAAAAAAC-3’ |
| RT-qPCR primers | MGMT Exp-F: 5’-CGAAATAAAGCTCCTGGGCA-3’  MGMT Exp-R 5’-GAACTCTTCGATAGCCTCGGG-3’ |


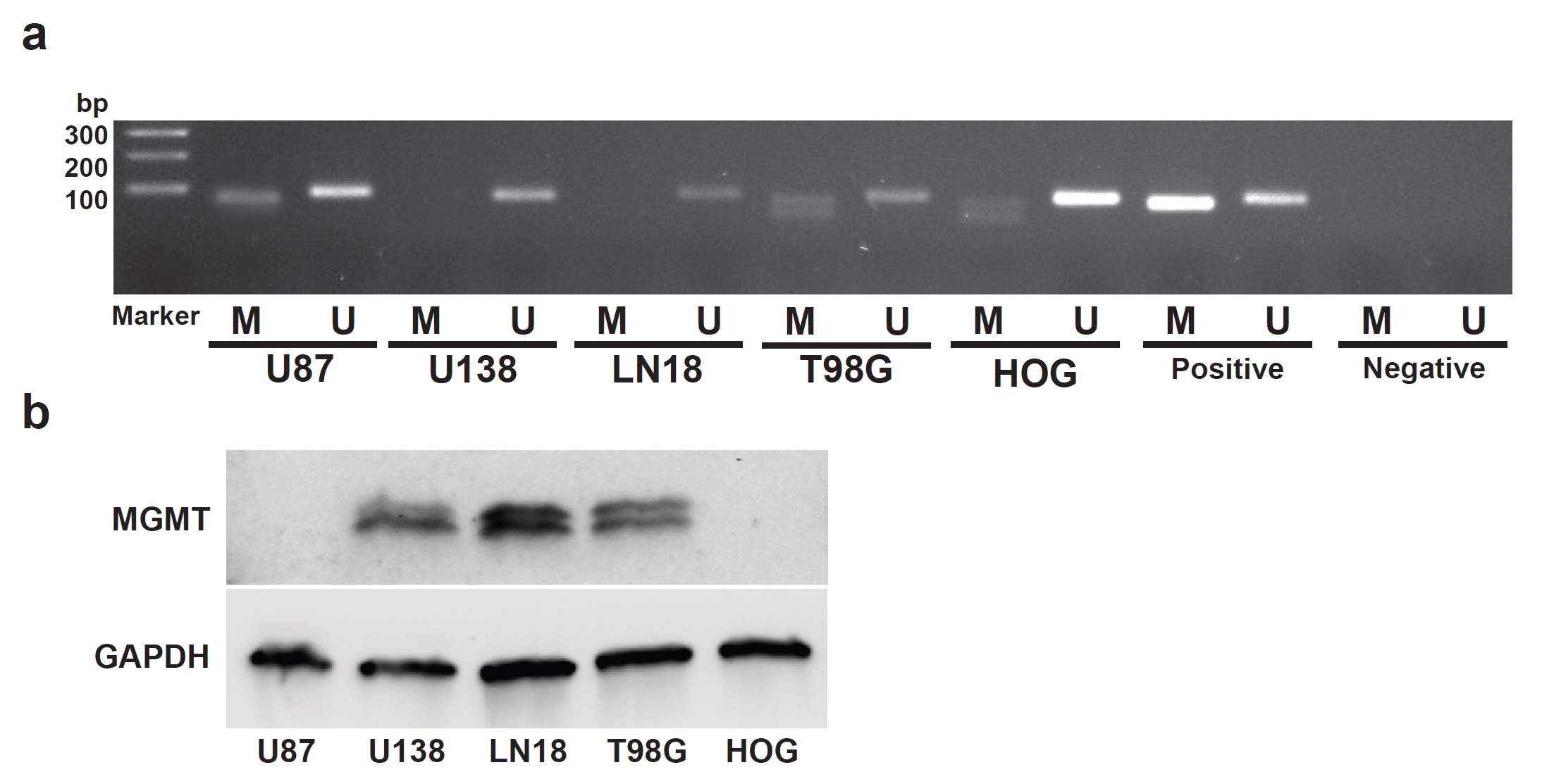


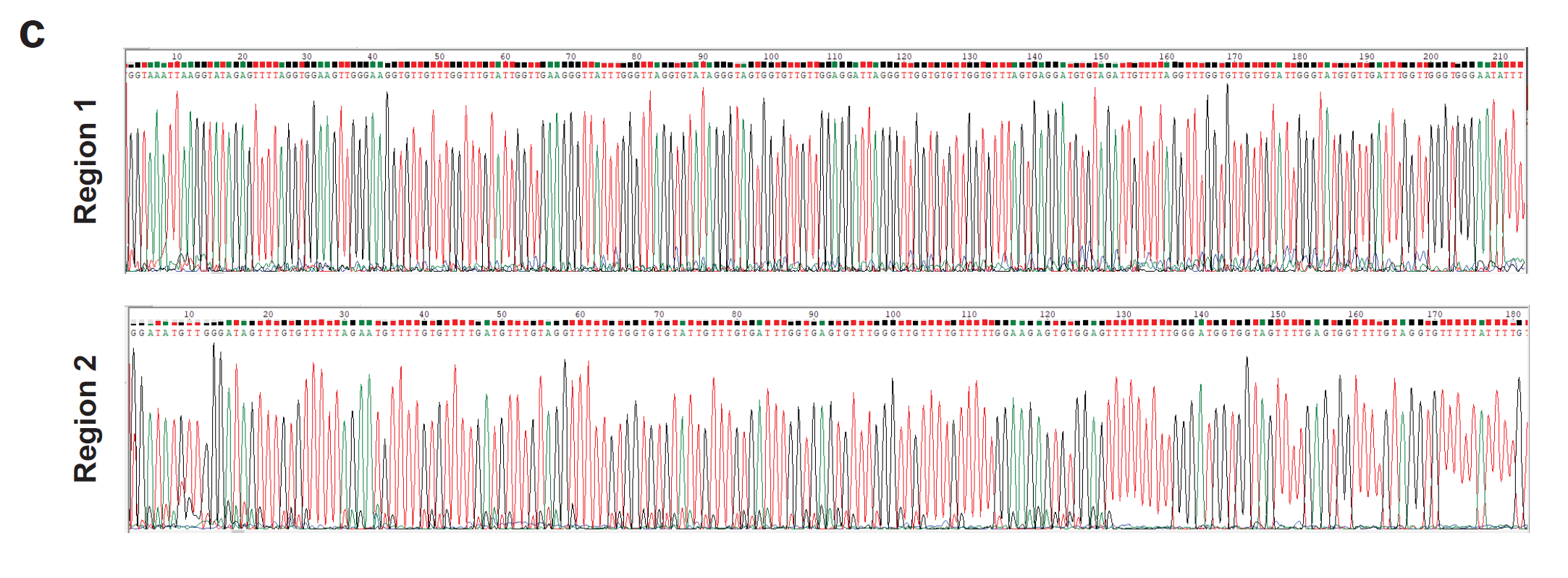


**Fig. S1 Initial screening verification of human glioma cell lines for unmethylated *MGMT***

**(a)** Methylation specific PCR (MSP) for *MGMT* gene: Gel electrophoresis. DNA was extracted from respective glioma cell lines as shown, or human oligodendroglioma cell line (HOG), treated with sodium bisulfite, and used for MSP analysis. Positive methylation control (Positive M) = whole genomic DNA treated *in vitro* with CpG methyltransferase *M.SssI*. Positive unmethylated control (Positive U) = Amplified whole genomic DNA *in vitro.* Negative M and U: PCR-grade H_2_O. **(b)** Western blot analysis showing MGMT protein expression levels in various glioma cell lines. Proteinlysates were generated from the same glioma cell lines as shown in Fig. S1a (human oligodendroglioma cell line also included). **(c)** Bisulfite sequencing of LN18 native glioma cells: Chromatograms demonstrate baseline unmethylated status of the *MGMT* gene. Region 1: *MGMT* region targeted by sgRNA 4 construct. Region 2: *MGMT* region targeted by sgRNA 1, 2, 3 constructs. DNA was extracted from native LN18 glioma cells to confirm baseline lack of methylation in the *MGMT* gene amplicons of interest, in the vicinity of the sgRNA constructs employed in the CRISPR-based d3A/sgRNA system. After sodium bisulfite treatment of DNA, to convert all cytosine bases to uracil, respective nested PCR primers were used to generate the amplicon regions of interest. Neither region exhibits any remaining cytosines in these amplicons, thus indicating lack of methylation at baseline.


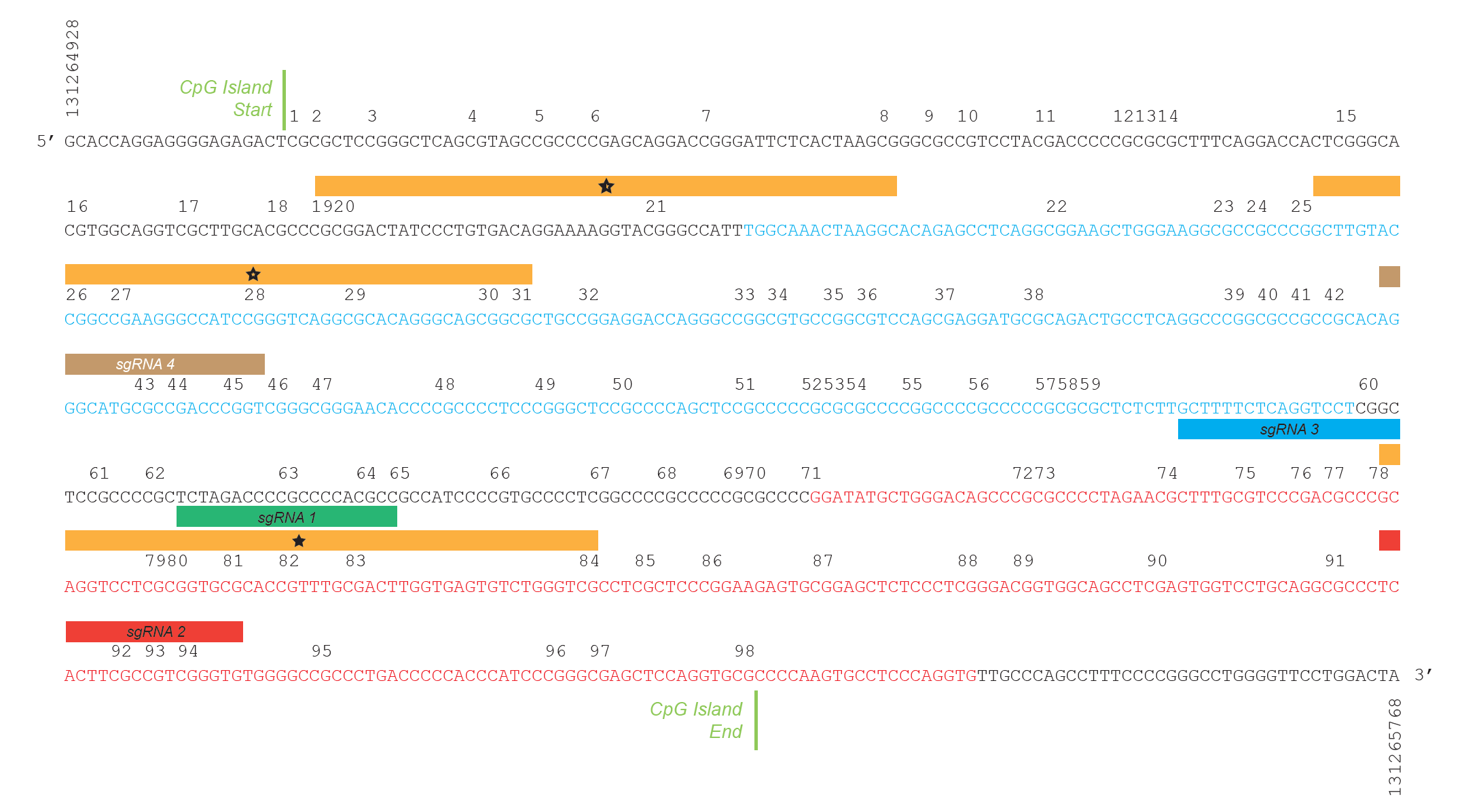


**Fig. S2 Complete sequence of the *MGMT* CpG island, with locations of island CpG sites, sgRNAs, key *Illumina* methylation probes, and nested PCR amplicons from bisulfite sequencing** Similar to the schematic shown in Fig. 1b but zoomed in further to specifically delineate the genetic sequences in relation to each sgRNA construct and the three differentially methylated *Illumina* probes identified, shown here is the native 5’ to 3’ sequence of the *MGMT* gene for GRCh37/hg19 chromosome 10 positions 131,264,928 to 131,265,768 on the forward DNA strand. The start and end points of the CpG island regions are indicated by the vertical green bars, and CpG sites in between these points are numbered from 1 through 98 in order of occurrence from 5’ to 3’. The orange bars indicate the span of differentially methylated *Illumina* probes, cg12434587 (open star) and cg12981137 (closed star), previously reported to be correlated with *MGMT* gene silencing and clinical outcome; we also identified a third probe, cg01341123 (half-closed star), differentially methylated. The brown, blue, green, and red bars indicate the loci of sgRNA4, 3, 1, and 2, respectively. Also highlighted are the locations of the two bisulfite amplicon regions for confirmation of CpG methylation at the single nucleotide level. The sgRNA4 amplicon region (Region 1) is highlighted by the blue sequence of nucleotides, while the sgRNA1, 2, and3 amplicon region (Region 2) is highlighted by the red sequence.


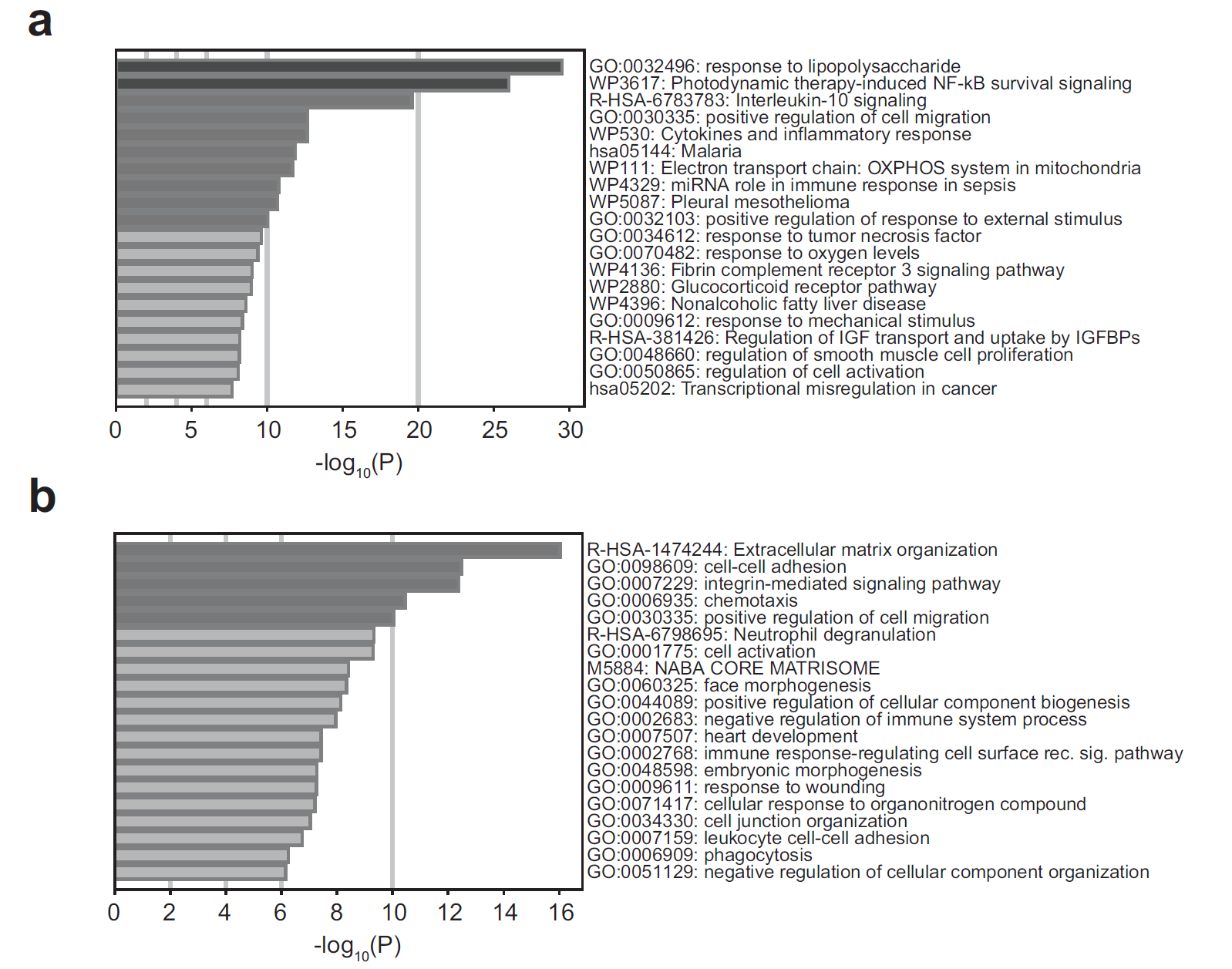


**Fig. S3 Metascape Gene Ontology (GO) enrichment analysis for differentially expressed genes obtained through RNA-Seq a)** Top 20 enriched terms obtained from Metascape for downregulated genes (DESeq2 log2 fold change < 0) between sgRNA and scRNA-treated LN18 cells as analyzed through DESeq2 (adjusted p-value < 0.05). Terms are listed in descending order by p-value.

**(b)** Similar to (a) but listing the top 20 enriched terms for upregulated genes (DESeq2 log2 fold change > 0).

**SUPPLEMENTARY INFORMATION: ADDITIONAL METHODS**

**Western blot analysis protocol: Additional details.**

Cells were lysed in 1x Pierce RIPA lysis buffer (*ThermoFisher,* Cat#89900) with proteinase inhibitor, 1:100 dilution (*ThermoFisher,* Cat#EO0491). Protein concentrations were measured using the Pierce BCA Protein Assay Kit (*ThermoFisher,* Cat#23225). Western blot was performed by standard protocols: Electrophoresis with 4–15% precast polyacrylamide gels (*Bio-Rad Laboratories*, Cat#4568084); TBST/5% dry milk was used as blocking buffer. Primary antibodies: anti-HA, rabbit (1:1000, *Sigma,* Cat#H6908-100mL); anti-MGMT, mouse (1:1000, *ThermoFisher,* Cat#35-7000); GAPDH, mouse (1:2000, *Proteintech* Cat#60004-I-Ig). Secondary antibodies: Goat anti-mouse IgG-HRP (1:5000) (*ThermoFisher,* Cat#62-6520); Goat anti-rabbit IgG HRP 1:5000 (*Abcam* #ab6721). Pierce^TM^ ECL Western Blotting Substrate kit (*ThermoFisher Scientific*, Catalog #32109) was used for HRP signal visualization. ChemiDoc XRS+ Imaging system (*BioRad*, Cat#1709690) was used to capture chemiluminescent images of immunoblot membranes.

**Differential methylation analysis and transcriptomic analysis: Detailed pipeline.**

***Illumina DNA analysis using linear fit models.*** Raw *Illumina* .idat data for all samples were imported into *R* [1] and normalized using the preprocessQuantile function (*minfi* package) in preparation for differential methylation analysis [2]. Data from probes with single nucleotide polymorphisms (SNPs) at CpG sites were omitted (dropLociWithSnps, *minfi*) to reduce the likelihood of false positives. Methylation raw data (M-values) were extracted for unsupervised hierarchical clustering and establishing methylation thresholds. Variance (standard deviation; SD) was calculated (using custom *R* scripts) per probe to determine M-value variability across the entire array, agnostic to cell sample origin. M-value data was sorted into two groups: sgRNA vs. scRNA. These data were fit to a linear model, contrasted based upon treatment (lmfit and contrasts.fit, *limma* package), and evaluated via empirical Bayes for differential methylation [3, 4]. Supervised hierarchical clustering was achieved by filtering M-value data based upon these summary statistics (p-adj < 0.05), as well as distributions of raw M-value data. Base methylation values from *Illumina* arrays tend to be bimodal in distribution [5]; values within range of the first peak (M-value < -1) were considered “low methylation”. Probes exhibiting M-value SDs more than 2 magnitudes above average array probe SD (M-value probe average SD > 0.7747001) were considered “variant” probes for purposes of determining On- and Off-target “hits”. Probes exhibiting M-value SDs more than 2.5 magnitudes above average were considered “highly variant” probes.

***Illumina bulk RNA-Seq analysis.*** Basecalled FASTQ data was aligned to the genome (GRCh37) using the command line tool *minimap2* with spliced variant mode enabled (-ax map-ont -uf -k14 –splice). This output (SAM) was converted to binary (BAM) and indexed using *samtools*. *HTSeq* was used to count genes (htseq-count –idattr=gene_id –type=exon –mode=union –format=bam –stranded=yes), which were imported into *R*, normalized, and formatted for DESeq2 differential expression analysis. Samples were compared based upon sgRNA treatment (sgRNA or scRNA); adjusted p-values (Wald test) were generated for each gene. Custom *R* scripts were written to cross reference these data with each CpG Island probe from *Illumina* 850K Methyl EPIC array DNA data, which could then be filtered and searched by DNA and RNA log-base 2 fold change in methylation or expression, adjusted p-values, M-value thresholds, etc. This intersectional data was used to validate “On-target” *MGMT* hits and “Off-target” hits in other genes that may have functional significance.

***Hypermethylated probes heatmap.*** CpG Island M-values were plotted as unsupervised and supervised hierarchical clustering heatmaps for data visualization. *Unsupervised*: CpG Island probes considered as “highly variant” (see above) were plotted to determine if cell samples naturally segregated by cell type (scRNA vs. sgRNA) and whether further supervised hierarchical clustering and analysis would be appropriate. *Supervised*: CpG Island probes were selected based upon eBayes differential analysis (adjusted p-value < 0.05) between NSC and sgR cells. Probes were further isolated based upon the following criteria: 1) Exhibit a low methylation value (M-value < -1) in control (scRNA) samples; 2) transition from a lower M-value (scRNA) to a higher M-value (sgRNA) to be considered “hypermethylated”; and 3) exhibit a degree of change larger than most probes across the full microarray (“variant” probe criteria as described above). This removed potential hits due to very small, but treatment-consistent, changes in methylation state with small p-values or methylation increases occurring at already highly methylated probes in control samples.

***RNA-Seq venn diagram and gene hits.*** *Illumina* probes were identified by gene name using a custom script to transform the “UCSC_RefGene_Name” splice variant values into gene identifiers. This was used to cross reference each *Illumina* probe to a *DESeq2* gene value. DNA differential methylation data (already filtered as described in “Hypermethylated Probes Heatmap” above) was further filtered by those that had differential RNA expression via *DESeq2* (Wald test p-adj < 0.05).

***Off-Spotter.*** “Off-Spotter”, a CRISPR designer tool provided by Thomas Jefferson University [6], was used to blast the sequences of sgRNA 1, 2, 3, 4 for hits. A search was performed for genome hits allowing up to 5 mismatches in the sgRNA sequences, with no fixed “seed” sequence (last 5 bases prior to the PAM at the 3’ end), and a variable PAM. These data were cross referenced to the *Illumina – RNA DESeq2 intersected* data by chromosome and position (GRCh37) to determine the shortest distance to a potential hit from each probe.

***Gene Ontology.*** Gene Ontology (GO) enrichment analysis was performed by feeding differentially expressed (DESeq2 adjusted p-value < 0.05) transcripts into Metascape [7] in two batches: One for downregulated genes (DESeq2 log2 fold change < 0) and one for upregulated genes (DESeq2 log2 fold change > 0).

***Programs used for schematics and heatmaps.*** Schematic artwork in this manuscript was generated using *R* and *Adobe Illustrator*. Heatmaps were generated via *pheatmap* and other figures using *ggplot2*.

**SUPPLEMENTARY INFORMATION: REFERENCES**

1. R Core Team (2014) R: A language and environment for statistical computing. R Foundation for Statistical Computing, Vienna, Austria. http://www.R-project.org/

2. Aryee MJ, Jaffe AE, Corrada-Bravo H, et al (2014) Minfi: a flexible and comprehensive Bioconductor package for the analysis of Infinium DNA methylation microarrays. Bioinformatics 30:1363–1369. https://doi.org/10.1093/bioinformatics/btu049

3. Ritchie ME, Phipson B, Wu D, et al (2015) limma powers differential expression analyses for RNA-sequencing and microarray studies. Nucleic Acids Research 43:e47. https://doi.org/10.1093/nar/gkv007

4. Smyth GK (2004) Linear Models and Empirical Bayes Methods for Assessing Differential Expression in Microarray Experiments. Statistical Applications in Genetics and Molecular Biology 3:. https://doi.org/10.2202/1544-6115.1027

5. Du P, Zhang X, Huang C-C, et al (2010) Comparison of Beta-value and M-value methods for quantifying methylation levels by microarray analysis. BMC Bioinformatics 11:587. https://doi.org/10.1186/1471-2105-11-587

6. Pliatsika V, Rigoutsos I (2015) “Off-Spotter”: very fast and exhaustive enumeration of genomic lookalikes for designing CRISPR/Cas guide RNAs. Biology Direct 10:4. https://doi.org/10.1186/s13062-015-0035-z

7. Zhou Y, Zhou B, Pache L, et al (2019) Metascape provides a biologist-oriented resource for the analysis of systems-level datasets. Nat Commun 10:1523. https://doi.org/10.1038/s41467-019-09234-6
